# Globalization, climate change, and transgenerational epigenetic inheritance: will our descendants be at risk?

**DOI:** 10.1186/s13148-014-0043-3

**Published:** 2015-01-22

**Authors:** Carlos Guerrero-Bosagna, Per Jensen

**Affiliations:** Avian Behavioral Genomics and Physiology Group, IFM Biology, Linköping University, Linköping, 58 183 Sweden

**Keywords:** Transgenerational epigenetic inheritance, Environmental exposures, Non-infectious diseases, Globalization, Industrialization, Food industry

## Abstract

Transgenerational epigenetic inheritance has gained increased attention due to the possibility that exposure to environmental contaminants induce diseases that propagate across generations through epigenomic alterations in gametes. In laboratory animals, exposure to environmental toxicants such as fungicides, pesticides, or plastic compounds has been shown to produce abnormal reproductive or metabolic phenotypes that are transgenerationally transmitted. Human exposures to environmental toxicants have increased due to industrialization and globalization, as well as the incidence of diseases shown to be transgenerationally transmitted in animal models. This new knowledge poses an urgent call to study transgenerational consequences of current human exposures to environmental toxicants.

## Transgenerational epigenetic inheritance

Transgenerational epigenetic inheritance has gained increased attention due to the possibility that exposure to environmental toxicants or other stressors can induce long-lasting changes in lineages of organisms [1-3]. The process involves germ line epigenomic changes that are transmitted to future generations and that associate with disease phenotypes [2,3]. Exposures to environmental toxicants such as fungicides, pesticides, or plastic compounds have been shown in animal models to produce abnormal reproductive or metabolic phenotypes that are transgenerationally transmitted. These include transgenerational increases in the incidence of obesity, polycystic ovary syndrome (PCOS), pregnancy defects, or germ cell apoptosis [4-8]. Importantly, the increased incidence of these transgenerationally transmitted diseases in response to environmental exposures in animal models is sometimes drastic. The current evidence on transgenerational epigenetic inheritance observed in animal models allows predicting that environmental exposures of today’s inhabitants of the world will strongly impact the incidence of non-infectious diseases in future generations. This would be correlated with long-lasting alterations in the epigenome of the gametes.

## Current human exposures

Human exposures to environmental toxicants have increased due to industrialization and globalization. The current state of globalization and climate change have helped the dispersion of toxicants in the environment by increasing the global transport of pollution, mobilization of legacy contaminants, and change in agricultural practices [9,10]. As result, it is expected that the amount and time that humans are exposed to environmental contaminants will increase even further, with unpredictable health consequences [9,10]. Moreover, increasing production and environmental accumulation of new compounds, initially produced to substitute previous persistent contaminants, is also occurring. These emerging contaminants have been found in all environmental compartments across the globe [11].

## Human exposures and diseases

A hidden aspect that has a fundamental impact on the life quality of the entire human population, and possibly its descendants, is exposure to environmental toxicants. Such exposure is hidden by the fact that most times one cannot be aware of our contact with these compounds, which are numerous and of increasing production and availability. Worldwide trends show association between environmental exposures and the incidence of non-infectious diseases [12]. Some of these diseases include the ones observed to be environmentally induced and transgenerationally transmitted in rodents, such as obesity, PCOS, or male fertility impairments [4-6,13,14]. For instance, in humans, obesity and overweight have experienced large increases from 1980 worldwide [15]. Such increase is proposed to be induced by changes in the environment [16]. PCOS is one of the main endocrine abnormalities in women, affecting around 7% of them and associating with reduced pregnancy, diabetes, obesity, and metabolic and cardiovascular diseases [17]. Although PCOS has been historically regarded as a genetic disease, recent evidence suggests that it would be mainly related to early developmental exposures [4], affecting a shared developmental pathway with metabolic diseases [17]. Another example is male reproductive function. Trends in human populations consistently show decreasing male reproductive parameters in the last decades [13,18]. Interestingly, many male reproductive disorders share a common developmental origin and patho-physiological etiology. These are grouped into the concept of ‘Testicular Dysgenesis Syndrome’, which would emerge due to environmental disruption during early testicular development [13,18]. Therefore, the common factor among these diseases is that they are environmentally induced during early development. Based on results in rodents, the current high incidences of these diseases in humans could be correlated to ancestral exposures to environmental contaminants such as DDT, BPA, phthalates, or hydrocarbon fumes [5,19].

Food consumption is an important route to environmental exposures. Recent practices in the food industry derived from globalization and industrialization have also been correlated with negative consequences for human health. Vastly used agro-compounds include fungicides or pesticides known to produce transgenerational epigenetic effects that include developmental, reproductive, and metabolic abnormalities [5,6,20]. Natural estrogenic compounds present in grains, i.e., phytoestrogens, also gain relevance nowadays, due to the widespread emergence of soy-based food for both human and farm animal consumption. Consumption of phytoestrogens is known to have epigenetic and reproductive effects [21]. On the side of animal-based food, the intensification of meat production in response to a growing world population, together with an increased demand for cheap food in large parts of the world, has caused large pressures on animal welfare on farms [22] and an increased exposure of humans to various pathogens, such as *Salmonella* and *Campylobacter* [23]. In addition, increased exposure to drug residues from preventive and other treatments of farm animals is also an issue of concern [23]. Disruption of the microbiome by inadvertent exposure to different chemicals emanating from the animal production industry may also affect health and behavior of humans in a range of as yet poorly investigated ways [24]. Most emphasis on food based exposures have been on agricultural compounds; however, emphasis should also be placed on animal conditions and treatments when considering human environmental exposures and the emergence of non-infectious diseases.

## Focus of policies on environmental epigenetics

Today we know that most non-infectious diseases are not explained by specific genetic variations but are rather related to environmental exposures during embryonic development or infancy [2]. Examples of these diseases include asthma, allergies, cancers, and obesity. Possibly, the widespread availability of environmental contamination and its future projections, together with the demonstrated biological possibility that these exposures can induce the onset of transgenerationally transmitted diseases, will have enormous effects in human health. Therefore, the new knowledge on environmentally induced transgenerational epigenetic inheritance should pose an urgent call for increased regulations on the production and use of environmental toxicants, as well as for research evaluating transgenerational effects derived from these exposures (in both humans and farm animals). It is becoming increasingly clear that the quality of life of our grandchildren depends on our current actions and exposures. In the same way, recent data strongly shows that many aspects of our health depend on what our grandmothers and grandfathers were exposed to in their lives.
